# Antibiotic-Loaded Nano-Sized Delivery Systems: An Insight into Gentamicin and Vancomycin

**DOI:** 10.3390/jfb15070194

**Published:** 2024-07-15

**Authors:** Silvia Pisani, Shafia Tufail, Mariella Rosalia, Rossella Dorati, Ida Genta, Enrica Chiesa, Bice Conti

**Affiliations:** 1 Department of Drug Sciences, University of Pavia, Via Taramelli 12, 27100 Pavia, Italy; silvia.pisani@unipv.it (S.P.); shafia.tufail@iusspavia.it (S.T.); mariella.rosalia01@universitadipavia.it (M.R.); rossella.dorati@unipv.it (R.D.); ida.genta@unipv.it (I.G.); enrica.chiesa@unipv.it (E.C.); 2 Department of Drug Sciences, IUSS Scuola Universitaria Superiore Pavia, 27100 Pavia, Italy

**Keywords:** nanomedicine, gentamicin, vancomycin, polymer nanoparticles, liposomes, antibiotic resistance, drug delivery

## Abstract

The fight against infectious disease has remained an ever-evolving challenge in the landscape of healthcare. The ability of pathogens to develop resistance against conventional drug treatments has decreased the effectiveness of therapeutic interventions, and antibiotic resistance is recognized as one of the main challenges of our time. The goal of this systematic review paper is to provide insight into the research papers published on innovative nanosized drug delivery systems (DDSs) based on gentamycin and vancomycin and to discuss the opportunity of their repurposing through nano DDS formulations. These two antibiotics are selected because (i) gentamicin is the first-line drug used to treat suspected or confirmed infections caused by Gram-negative bacterial infections and (ii) vancomycin is used to treat serious Gram-positive bacterial infections. Moreover, both antibiotics have severe adverse effects, and one of the purposes of their formulation as nanosized DDSs is to overcome them. The review paper includes an introduction focusing on the challenges of infectious diseases and traditional therapeutic treatments, a brief description of the chemical and pharmacological properties of gentamicin and vancomycin, case studies from the literature on innovative nanosized DDSs as carriers of the two antibiotic drugs, and a discussion of the results found in the literature.

## 1. Introduction

**Figure 1:**
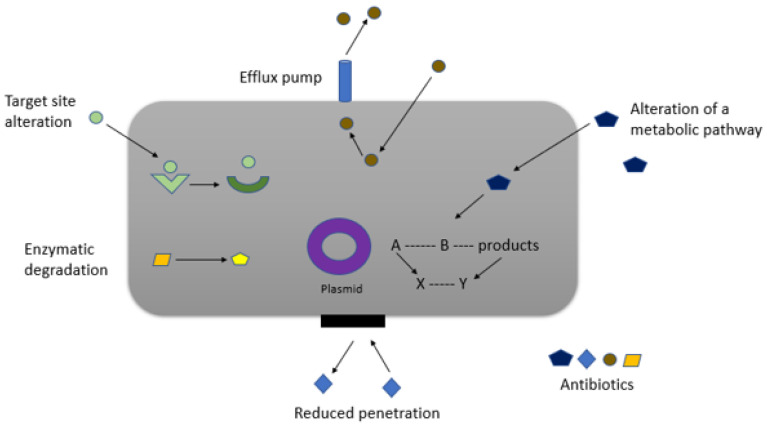
Scheme showing different mechanisms of antibiotic resistance.

The fight against infectious disease has remained an ever-evolving challenge in the landscape of healthcare. The ability of pathogens to develop resistance against conventional drug treatments goes through various mechanisms as shown in Figure 1, and it has decreased the effectiveness of therapeutic interventions. Thus, it is necessary to develop innovative strategies in drug delivery to combat the complexities of microbial resistance. The World Health Organization (WHO) updated the Bacterial Priority Pathogens List (BPPL) 2024, including 15 families of antibiotic-resistant bacteria classified into critical, high, and medium categories for prioritization. The BPPL addresses current challenges and provides essential guidance for policymakers, national health authorities, and others involved in decisions about the R&D of new antibiotics and treatments and investment. Compared with the 2017 list, the dynamic nature of antimicrobial resistance (AMR) necessitated implementations. Leveraging the BPPL as a global tool, customizing the list to fit country and regional contexts, can accommodate variations in pathogen distribution and the AMR burden [1].

This introductory part will provide a comprehensive overview, emphasizing the significance of anti-infective drug delivery, the challenges linked with traditional methods, and the importance of gentamicin and vancomycin in battling infectious threats [2].

### Overview of the Significance of Anti-Infective Drug Delivery

The delivery of anti-infective drugs represents a critical aspect in developing effective and targeted therapies against microbial infections. The ability of anti-infective drug delivery extends beyond the boundaries of traditional treatments, resulting in a paradigm shift in how we can tackle infectious diseases. Unlike non-infectious diseases, the fluctuating and adaptive nature of pathogens requires a site-specific drug-delivery approach, one that provides optimal drug concentrations at the site of infection while reducing systemic side effects [3].

The need to focus on more performing anti-infective drug delivery is based on the fact that gaining therapeutic success goes beyond the mere potency of the drugs themselves. The complex interplay of host–pathogen interactions, the immune system, and medication pharmacokinetics need a personalized and precise approach to administration. An anti-infective drug delivery strategy aims to increase the bioavailability of therapeutic agents, prolong efficacy, and overcome the barriers posed by the blood–brain barrier (BBB) or biofilm formations, which represent the common obstacles to infectious disease treatment [4]. Thus, to overcome the global surge in antibiotic resistance and the drawback of systemic administration’s inability to maintain optimal drug concentrations at the infection site, it is necessary to exploit different delivery strategies, such as targeted and/or localized drug delivery. In this context, the strategic use of nano drug-delivery platforms enables targeted drug delivery, minimizing the risk of resistance development and maximizing the therapeutic impact. Wide literature is available on this topic because nowadays it is a hot topic for health and healthcare management [5].

The goal of this review is to provide insight into the research papers published on innovative nanosized drug delivery systems based on gentamycin and vancomycin and to discuss the opportunity of their repurposing through nano drug delivery system formulations. These two antibiotic drugs have been selected because (i) gentamicin is the first-line drug used to treat suspected or confirmed infections caused by Gram-negative bacteria and (ii) vancomycin is an example of an antibiotic drug used to treat serious Gram-positive bacterial infections.

## 2. Challenges Associated with Infection Therapies Based on Traditional Drug Formulations

Traditional drug formulations, while useful in many therapeutic areas, present significant obstacles when applied to anti-infective medicines. Antibiotics used systemically often result in insufficient drug concentrations at the infection site, necessitating greater doses, which can contribute to systemic toxicity. Furthermore, variability in patient responses, driven by factors, such as the immunological status and comorbidities, hampers the predictability of therapy outcomes [2].

As you look at the variety of infections, each of which presents distinct obstacles in terms of drug delivery, the need for creative new and more performing strategies becomes necessary. For example, treating biofilm-associated conditions is challenging due to the protective shield provided by biofilm matrices. Microbial biofilms are complex microbial communities encased in extracellular polymeric substances (EPSs) that confer adaptive resistance and physical protection to the cells within. Biofilms can be composed of a single microorganism or a mixture of bacteria, fungi, archaea, protozoa, and yeasts. The three-dimensional structure of a biofilm encompasses channels that control the release of gases and nutrients. Biofilms can be up to 5000 times more tolerant to antibiotics than planktonic bacterial cells and are often associated with chronic infections. Since biofilms are rarely completely eliminated, even after prolonged treatment with antibiotics, they can recur after a period of clinical quiescence and present characteristics of greater resistance to traditional therapies.

One of the most frequent conditions is the medical device-related biofilm that is rising as an infection issue due to the widespread use of medical devices implanted in the human body (i.e., prosthesis). Free-floating bacterial cells can aggregate to form biofilms on the implanted medical device surface, and this poses a severe threat to the life and health of patients. Device-associated infections usually occur during treatment, where some microorganisms originate from the host. The pathogenesis of medical-device-associated infections is related to microorganisms in complex communities that adhere to and grow on device surfaces forming a biological container. Medical device-related biofilms can consist of single or multiple species, depending primarily on the type of device and the time it is left in the patient’s body.

Intracellular pathogens, such as *Mycobacterium tuberculosis*, *Salmonella* spp., and *Francisella tularensis*, are especially challenging to be eradicated since they invade host cells and survive inside them. This infecting behavior protects the intracellular pathogens from both antibiotics and the host immune systems, making them extremely recalcitrant to being completely eradicated. Moreover, the infected cells can act as “Trojan horses”, delivering the bacteria to noninfected tissues, and this mechanism contributes to treatment failure and recurring infections.

These challenges highlight the demand for innovative therapeutic delivery techniques able to cross cellular barriers and reach the infection’s hidden reserves [6,7]. Nanoparticle-based techniques provide a varied and adaptable response to these issues. Nanoparticles’ unique physicochemical features, such as size, surface charge, and biodegradability, can be used to optimize drug delivery for certain illnesses. These platforms enable the encapsulation of anti-infective drugs, preventing degradation, facilitating controlled release, and increasing bioavailability at the target site [8]. Innovation in drug administration also addresses the essential issue of patient therapeutic adherence, which is a major concern in infectious disease management. Traditional oral antibiotic regimens frequently require strict adherence to prescribed schedules, which can be difficult for patients, especially in resource-limited settings. Nanoparticle-based therapy, with the possibility for extended-release and a reduced dose frequency, provides a more patient-friendly option, potentially enhancing treatment results and lowering the risk of resistance through improved adherence due to less frequent dosing, which makes treatment easier to follow [9].

## 3. Gentamicin and Vancomycin

Antibiotics represent a crucial weapon in the constant battle against bacterial infections. Among these, gentamicin and vancomycin occupy a prominent position, thanks to their unique properties and role in fighting against various pathogens. However, due to the growing incidence of antibiotic resistance, their effectiveness is exposed to serious risks, resulting in some cases being ineffective in eradicating infections.

### 3.1. Gentamicin

**Figure 2:**
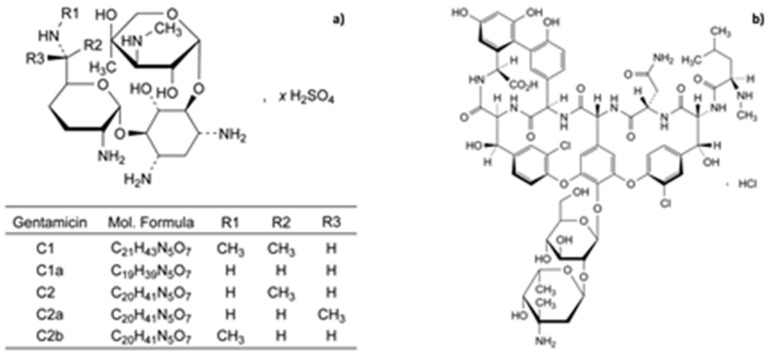
Chemical structure of (**a**) gentamicin and (**b**) vancomycin (modified from European Pharmacopeia 11th Ed) [11].

Gentamicin is a member of the aminoglycoside class of antibiotics. It was isolated in 1963 by Weinstein and colleagues from the soil fungus Micromonospora purpura (of the Actinomycete group). It was introduced in the USA in 1969. It is a “complex” of gentamicin’s C_1_, C_1a_, and C_2_ and also gentamicin A, which differs from the other members of the complex but is similar to kanamycin C. Figure 2a shows the gentamicin C sulfate chemical structure as reported in the European Pharmacopeia. The different gentamicin conformers are basically due to diverse R1, R2, and R3 substitutions. The two amino sugars joined in a glycosidic linkage to a hexose nucleus (2-deoxystreptamine) make gentamicin an aminoglycoside aminocyclitol. It is a highly water-soluble polar cation at pH 6–8, while it is moderately soluble in ethanol, methanol, and acetone and insoluble in benzene and halogenated hydrocarbons. It melts with decomposition in the range of 200–250 °C. Gentamicin antibiotic activity is inhibited by acid pH and divalent cations.

**Figure 3:**
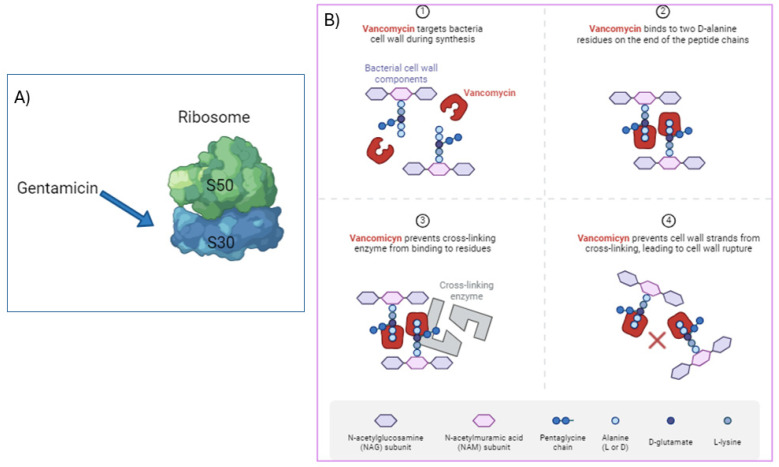
Schematized mechanisms of action of (**A**) gentamicin and (**B**) vancomycin. **X** in panel 4 highlights Vancomycin prevents cell wall strands from crosslinking (done with Biorender).

Gentamicin is effective against both Gram-positive and Gram-negative organisms and particularly useful for the treatment of severe Gram-negative infections. It works by irreversibly inhibiting the protein synthesis essential for bacterial cell survival. Gentamycin specifically binds to the 16S ribosomal RNA aminoacyl site of the 30S ribosomal subunit, which is responsible for protein translation (Figure 3A). This binding interferes with the formation of peptide bonds, leading to non-functional and incomplete protein synthesis, ultimately killing the bacteria [10].

The primary application of gentamycin involves treating serious infections caused by Gram-negative bacteria, such as *Pseudomonas aeruginosa*, *Klebsiella pneumoniae*, and *Escherichia coli*. It is often employed as a last-line therapy against multidrug-resistant bacteria because of its broad-spectrum activity. Gentamycin is mostly administered intravenously or intramuscularly, with the dosage and duration tailored to the specific infection and patient profile [12]. For example, in bacterial *Peritonitis*, gentamicin is used for the short-term prevention and treatment of soft tissue infection associated with abdominoperineal resections or operations on the small or large bowel [13]. The usual adult dose for systemic infections is 1 mg/kg IM or IV infusion every 8 h. Also, in *Pseudomonas aeruginosa* infections, gentamycin is effective against these severe Gram-negative infections. The usual adult dose for life-threatening infections is an initial dose of 5 mg/kg IM or IV infusion per day, given in divided doses three to four times a day [14]. The peak serum concentrations of gentamicin are reached after 30 to 90 min of administration via the parenteral route. The molecule is polar and water soluble, and thus, its distribution in the central nervous system and general cells is low, while the eight cranial nerves (vestibular area of hear) and kidneys are its target organs.

As far as renal toxicity is concerned, it should be mentioned that most gentamicin is excreted unmetabolized via glomerular filtration, which enables a urinary concentration, in the renal cortex, almost 100-fold higher than the serum. This mechanism makes the kidney one of the target organs of the drug, and, due to the extremely high concentration reached in kidneys, reversible renal damage can develop in terms of mild proteinuria and a reduction in the glomerular filtration rate. Toxic effects can also develop in the vestibular area, leading to deafness. The damage to the vestibular portion of the eighth cranial nerve appears to be greater than that to the cochlear portion. This often appears with high-pitched tinnitus. Ototoxicity is more frequent with long-term gentamicin therapy.

The co-administration of gentamicin together with other antibacterials, such as beta-lactams, for example carbenicillin, shows a synergistic effect to treat infections caused by *Pseudomonas aeruginosa*. The synergistic activity is not only important for the treatment of complex infections but can also contribute to dose optimization and reduced adverse effects. Gentamicin is eliminated mainly via renal excretion with a mean half-life of 75 min after intravenous administration and 104 min after oral administration. Because of the gentamicin renal excretion prevalence, a low rate of creatinine clearance (renal impairment) is associated with a longer gentamicin half-life. Some pathologic conditions, such as fever, anemia, and severe burns, may result in a transient shorter half-life and lower gentamicin serum concentrations. Since gentamicin is distributed in extracellular fluids, a change in the body fluid balance and increased metabolism rate caused by these pathological states can affect gentamicin serum levels and the half-life.

Some substances, such as chondroitin sulfate, may decrease the excretion rate of gentamicin raising its serum level [15].

Bacteria can develop resistance to gentamicin through various mechanisms, such as enzymatic modification of the drug, mutations in the ribosomal binding site, and efflux pumps that expel the antibiotic, as depicted in Figure 1. This resistance primarily concerns Gram-negative bacteria, such as *Pseudomonas aeruginosa*, where multidrug resistance is increasingly prevalent [16].

### 3.2. Vancomycin

Vancomycin was isolated in 1956 from *Amycolatopsis orientalis* (also known as *Streptomyces orientalis*, *Nocardia orientalis*). Vancomycin is stable at a pH range of 3–5 and usually stored at 2–8 °C. However, its stability can be dependent on factors, such as the temperature, concentration, and pH [17]. It belongs to the glycopeptide antibiotic class; its structure consists of a seven-membered peptide chain forming a tricyclic ring system to which the disaccharide formed by vancosamine and glucose is linked (Figure 2b). The *N*-terminal amino acid leucine is critical for vancomycin’s antibacterial activity, which is known for its effectiveness against Gram-positive bacteria, particularly methicillin-resistant *Staphylococcus aureus* (MRSA). It particularly inhibits cell wall synthesis in bacterial cells. Vancomycin’s mechanism of action resides in its binding to the D-alanyl-D-alanine residues, the building blocks of the peptidoglycan layer, preventing the formation of cross-links essential for cell wall integrity. This weakened cell wall leads to bacterial death or lysis (Figure 3B) [18].

Vancomycin is a critical lifeline for patients with infections caused by resistant Gram-positive bacteria, including MRSA and *Vancomycin-resistant Enterococcus* (VRE). It is often used as a last resort where other antibiotics have failed. It is also used for the treatment of Clostridium difficile-associated diarrhea and enterocolitis caused by *Staphylococcus aureus*, including methicillin-resistant strains. Vancomycin is generally administered intravenously, and because its oral absorption is poor, it is orally administered only to treat intestine infections. It is eliminated in 24 h mainly through kidney excretion with a bi-phasic elimination half-life, with the initial half-life being relatively quick and a terminal half-life of 4 to 6 h in healthy adults with a normal renal function. The elimination half-life is significantly prolonged in patients with renal dysfunction. The dosage and therapy duration should be repeatedly monitored due to potential kidney toxicity [19,20].

Vancomycin resistance is comparatively less common as compared to other antibiotics; still, the cases of MRSA and VRE infections are gradually increasing. This creates a significant threat, as treatment options become limited for such infections. The mechanisms of vancomycin resistance include modification of the cell wall target and acquisition of alternative cell wall synthesis pathways [21].

### 3.3. Beyond Resistance: Other Concerns and Strategies for Sustainable Use

Both gentamicin and vancomycin show a limited spectrum. Gentamicin primarily targets Gram-negative bacteria, while vancomycin focuses on Gram-positive pathogens. This limitation requires the use of additional antibiotics to cover other bacterial strains in mixed infections [22]. Strategies for the sustainable use of these antibiotics and in general for improving antibiotic therapy can be listed. First of all, the judicious use of antibiotic drugs and their utilization only when necessary and for the shortest effective duration is recommended to minimize selective pressure for resistant bacteria. Developing new antibiotics, particularly those targeting resistant bacteria, or combining multiple therapies, is critical to maintain a robust strategy against evolving pathogens and enhance effectiveness to delay the emergence of resistance. Moreover, rapid and accurate diagnostic tools to identify the specific bacterial pathogen and its resistance profile are significant for deciding on the appropriate antibiotic therapy to act promptly on the infection.

Vancomycin and gentamicin are an example of a perfect combination for a dual approach in anti-infective therapy due to their complementary modes of action. The goal is to establish a synergistic platform that addresses a wide range of bacterial illnesses, decreasing the danger of resistance development and maximizing therapeutic outcomes by combining their capabilities within nanodrug delivery systems [23]. The importance of the anti-infective drug delivery strategy, highlighted by the difficulties associated with conventional techniques, and the indispensable function of drugs, such as vancomycin and gentamicin, paves the way for an extensive investigation of nanoparticle-based strategies in the sections that follow in this review.

## 4. Rationale for Nanoparticle-Based Drug Delivery

**Figure 4:**
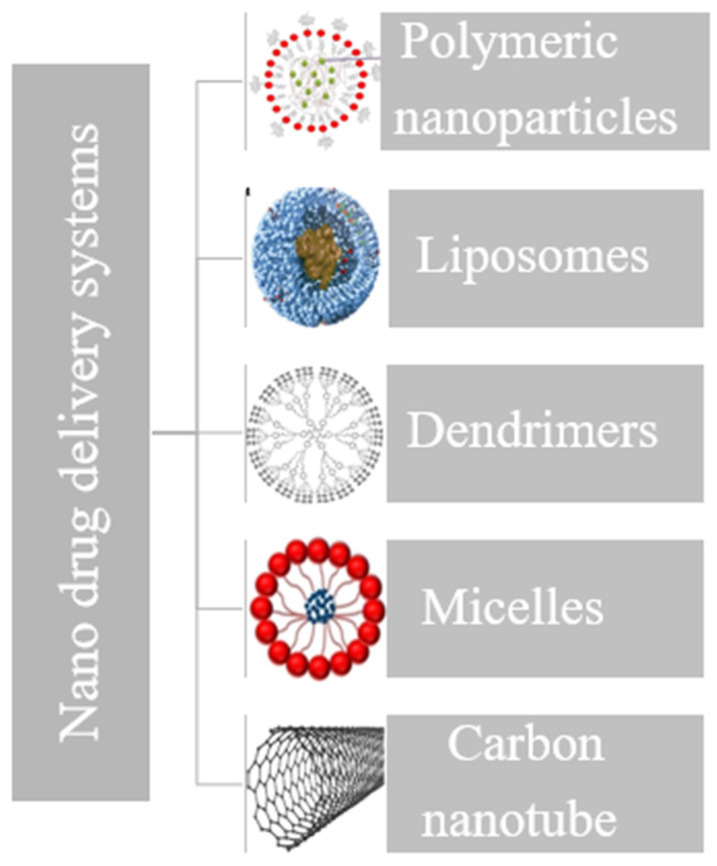
Nanosized drug delivery systems proposed as antibiotic carriers.

The combination of nanoparticles with antibiotics, either through encapsulation or conjugation, has been thoroughly studied, and still it is because nanosized drug delivery systems offer considerable advantages over traditional drug formulations. Different types of nanosized drug delivery systems have been reported in the literature as carriers for antibiotics. As shown in Figure 4, they have various morphologies, also depending on their composition.

The Web of Sciences (WOS) reports 16,527 papers published on this topic up to today (May 2024). In general, among the advantages of nanoparticulate carriers, the following should be mentioned: (i) the ability to offer controlled and prolonged drug release, allowing the drug to remain in the therapeutic window in the body for a longer time, improving therapeutic effectiveness while also reducing the frequency of drug administration, thus increasing patient compliance [24]; (ii) the possibility of achieving target delivery through nanoparticle engineering with ligands on its surface, which can specifically bind to receptors on target cells. This increases treatment efficacy by ensuring that the drug is mainly delivered where it needs to be administered, while also reducing side effects by limiting drug exposure to non-target tissues [8].

**Figure 5:**
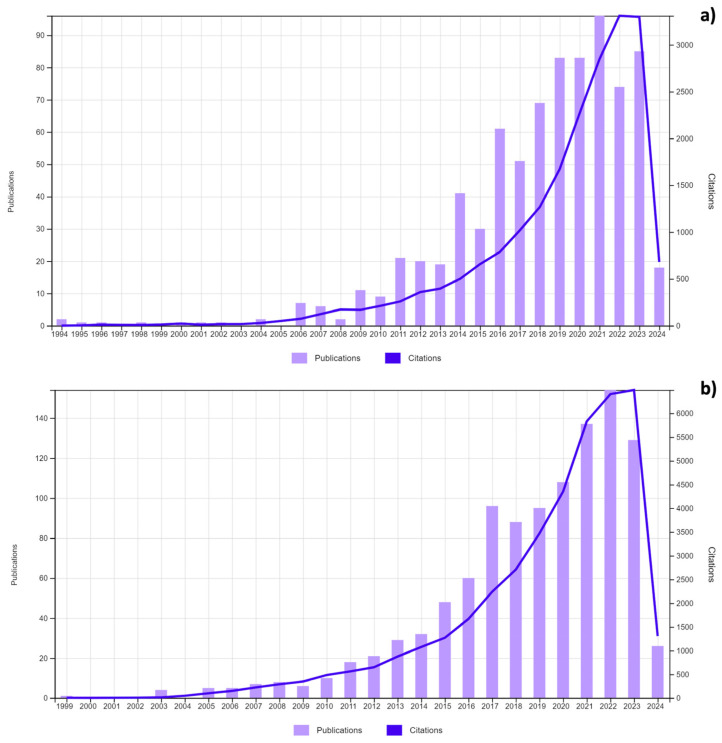
Number of publications and citations on (**a**) gentamicin nanoparticles and (**b**) vancomycin nanoparticles in the time range 1985–2024. From the WOS citation report, April 2024.

As far as antibiotics are concerned, nanosized drug delivery systems can increase antibiotic bioavailability through an increase in the apparent drug solubility (a higher surface-area-to-volume ratio permits more drug molecules to be exposed to the corresponding medium), membrane permeation, and antibiotic stability, ensuring that a greater proportion of the medicine reaches its intended site of action. This is especially useful for drugs, such as gentamicin and vancomycin, which are poorly absorbed (less than 1% of the dose is absorbed following oral or rectal administration) in the body [25,26]. Thus, nanoparticles as carriers for gentamicin and vancomycin have been investigated mainly in the last 10 years, as reported by the WOS citation report and shown in Figure 5; 796 and 1087 papers have been published, respectively, on gentamicin and vancomycin encapsulation in nanosized drug delivery systems in the time range between the years 1985 and 2024.

The specific advantages of nanoparticles as carriers for gentamicin and vancomycin can be highlighted as follows: (i) protection from antibiotic degradation (the slow release of the antibiotic prevents it from immediate exposure and degradation), (ii) ability to reduce antibiotic toxicity, and (iii) to overcome antibiotic resistance [27].

Table 1 and Table 2 report some examples of experimental works on various nanosized delivery systems loaded with gentamicin and vancomycin, highlighting the material nanoparticles are made of and their preparation techniques. The majority of examples found in the literature refer to polymer nanoparticles loaded with gentamicin or vancomycin, the polymers being either synthetic, such as poly-alfa-hydroxyacids, or natural, such as chitosan. The most frequent techniques reported to prepare the polymer nanoparticles loaded either with gentamicin or vancomycin are the traditional ones, i.e., emulsion solvent evaporation (or double emulsion solvent evaporation) when synthetic biodegradable polymers, such as polylactide-co-glycolide (PLGA), polyethylenglcol-co-polylactide-co-glycolide (PEG-PLGA/PLGAH) or polyurethane, are used as carriers, and ionotropic gelation when polymers of a natural origin, such as chitosan, are used as carriers. Both antibiotics have also been combined with inorganic nanoparticles made of silver, silica, and iron that can be external stimulus-responsive carriers. Nanosized systems loaded with gentamicin or vancomycin were tested on popular bacteria, such as *Staphylococcus aureus* (*S. aureus*), *Escherichia coli* (*E. coli*), and *Pseudomonas aeruginosa* (*P. aeruginosa*). As said, these are taken as an example of very frequent and severe infections from Gram-positive bacteria (i.e., methicillin-resistant *Staphylococcus aureus* (MRSA)) and Gram-negative infections (i.e., *P. aeruginosa* in patients affected by cystic fibrosis) that are difficult to be treated and that show a high tendency to eradicate.

## 5. Gentamicin (GS) and Vancomycin (VM) Nanosized Delivery System Case Studies

### 5.1. Polymeric Nanoparticles

Polymer nanoparticles are submicron-sized polymeric colloidal particles, with sizes ranging from 10 to 200 nm. They are generally composed of natural or synthetic polymers and can encapsulate a range of drugs, including anti-infective agents, such as gentamicin and vancomycin [58].

A wide range of polymeric nanoparticles has been studied and developed to deliver antibiotics. They have shown promising results in preclinical and clinical studies, proving their potential for drug delivery. As reported in Table 1, various authors investigated the encapsulation of gentamicin sulfate into polymer nanosized drug delivery systems. The papers found in the literature report three main strategies: (i) encapsulation of GS into polymer nanoparticles, (ii) encapsulation of gentamicin into nanofibrous patches, and (iii) loading GS and/or GS NPs into nanofibrous patches. The antimicrobial effect of GS-loaded nanosized drug delivery systems is always tested towards *P. aeruginosa* and *S. aureus*, which are among the most frequently identified pathogens towards which GS is active. As far as drug release is concerned, the main mechanism of GS release from polymer nanoparticles relies on the Higuchi kinetic model based on Fickian diffusion. A burst release, accounting for 20–40% of GS release in the first hour of incubation is highlighted, due to the amount of drug closer to the NP surface and depending on the polymer matrix composition.

For example, Y. Sun and colleagues and R. Dorati and colleagues investigated the encapsulation of GS in nanoparticles made from polylactide-co-glycolide (PLGA), a poly-alfa-hydroxy acid whose biocompatibility and biodegradability is well known and approved for human use by international regulatory agencies. Y. Sun and colleagues [38] focused their work on the nanoparticle preparation process using plain PLGA and a conventional double emulsion solvent evaporation method to prepare GS-loaded PLGA nanoparticles. They extensively studied the process variables and concluded that PVA and PLGA concentrations were critical factors in determining the particle structure and size. The NP size increased up to the micron size when the PVA concentration increased, and GS release was affected by the particle porosity. Therefore, the gentamicin-loaded PLGA nanoparticles could be tuned using the double emulsion evaporation method with different parameters, including the PVA (surfactant) concentration and PLGA concentration, resulting in effective antibacterial activity.

Dorati and colleagues [43] carried out a detailed study on a gentamicin-loaded biodegradable nanoparticle (NP) formulation using modified PLGA polymers, such as uncapped polylactide-co-glycolide (PLGA-H) and polylactide-co-glycolide-co-polyethylenglycol (PLGA-PEG) blends; a solid-oil-in water technique was used as an NP preparation method. The work was focused on a screening design to optimize the drug payload, NP size and size distribution, NP stability, and resuspension after freeze-drying. The authors concluded that the particle size and drug content (DC) were mostly affected by the polymer concentration. By studying the experimental parameter through a 2^3^ screening design, i.e., the polymer blend composition (PLGA-PEG and PLGA-H), polyvinylalcohol (PVA), and methanol concentrations into the aqueous phase, they were able to increase the drug content up to 10.5 *w*/*w*%. The stirring rate resulted in the most influencing factor for the size distribution (PDI): 700 rpm permitted a homogeneous NP dispersion (PDI = 1) with an average NP size of 200 nm to be obtained. Nanoparticle lyophilization was studied by adding cryoprotectants, polyvinypirrolidone K17 and K32, and sodium carboxymetylcellulose. The freeze-drying protocol was optimized through a mixture design, obtaining a free resuspendable freeze-dried powder made from stable NPs with a suitable size and payload. The powder was tested on clinical bacterial isolates demonstrating that after encapsulation, gentamicin sulfate kept its activity. Moreover, the authors carried out a kinetic study on in vitro GS release from the NPs highlighting that GS release follows the Higuchi model with a release Fickian diffusion mechanism.

The interest in loading GS into polymer patches, namely nanofibrous polymer ones, has been demonstrated in several works. Another example is the work of Pisani and colleagues They investigated polylactide-*co*-polycaprolactone electrospun nanofiber matrices as a carrier for GS demonstrating prolonged drug release and an increase in its antimicrobial effect. Electrospun matrices can be used on severe burns to prevent infections, implanted into gingival cavities for local infection treatment, or applied after tooth extraction. Their advantage lies in a controlled delivering of high antibiotic concentrations directly to the site of action while minimizing systemic concentrations, thereby reducing drug side effects [59]. Y. Sun and colleagues loaded the PLGGA-loaded GS NPs with size of 130 nm into nanofibrous polyurethane patches via electrospinning obtaining wound-healing patches with antibacterial activity. The paper demonstrates the ability of this drug delivery system to slow down the release of GS from NPs embedded into the nanofibers, by keeping their antibacterial effect [42]. The key to the incorporation of the NPs into the nanofiber scaffolds lies in the process of electrospinning and the properties of the other materials involved. The authors synthesized the GS-loaded PLGA NPs via a double emulsion solvent evaporation method, as reported above. During electrospinning, the solvent (DCM) rapid evaporation causes the polymer (PU/PEO) to solidify, encapsulating the PLGA NPs within the nanofiber scaffolds. The authors found that the purified PLGA NPs were uniformly and individually incorporated into the PU/PEO nanofiber scaffolds. The presence of PEO in the mixture significantly improved the compatibility of PLGA NPs and PU, resulting in a well-dispersed distribution of PLGA NPs in the nanofiber scaffolds. This suggests that the PLGA NPs, despite PLGA being soluble in DCM, can be successfully incorporated into the nanofiber scaffolds due to the rapid solidification of the PU/PEO during electrospinning and the compatibility among PLGA, PU, and PEO.

Dhal and colleagues [36] investigated GS-loaded PLGA nanoparticles loaded into pullulan films (PNP-F) for the treatment of a nosocomial infection or surgical site infection. The author’s focus was on the sterilization of PNP-F with EtO treatment. They demonstrated that EtO treatment did not cause any effects in the in vitro disintegration time, % of drug loading, and antimicrobial effectiveness, but led to a change in the PNF-F mechanical properties due to the plasticization effect. Moreover, PNP-F was stable at 25 ± 2 °C/RH 60 ± 5% storage conditions for 3 months only; thus, 15 °C was proposed as the storage temperature, and the authors also suggested controlled humidity storage conditions, because of the deliquescent nature of GS. GS nanoparticles embedded in the pullulan films resulted in slow GS release, up to 192 h, and the wound healing assay confirmed the PNP-F effectiveness towards a fibroblast cell line (NIH-3T3) in facilitating the growth and inhibition of colonies of *P. aeruginosa* and *S. aureus.* In vivo studies indicated faster healing without scar formation in incisions receiving PNP-F compared to marketed GS cream and untreated incisions. Thus, the authors concluded that PNP-F can be explored as an alternative for the management of nosocomial infections or surgical site infections.

Gentamicin has also been formulated into nanosized drug delivery systems made from chitosan, also exploiting the antibacterial properties of this natural polymer. Simpson and colleagues formulated stable chitosan and TPP particles capable of loading various GS concentrations up to 65% and whose size ranged from 100 to 400 nm, with PDI (less than 0.5) and negative zeta potentials. In vitro antimicrobial release studies against *P. aeruginosa* and MRSA demonstrated effectiveness, with up to 90% release over 7 days, achieving significant bacterial reduction within 3 h for the formulation with the highest drug concentration. Comparative efficacy analysis revealed promising results compared to existing formulations. These findings indicate potential applications in enhancing bone healing and preventing or treating infections, either through incorporation into scaffolds or hydrogels or as standalone treatments. Further research, especially in vivo studies, is necessary to validate and expand on these promising results [60].

Concerning VM, a lower number of papers have been found in the literature about vancomycin encapsulation in polymer nanoparticles.

An interesting work on VM encapsulation was written by Cerchiara and colleagues about VM-loaded chitosan nanoparticles embedded in Spanish broom fibers making a wound dressing. The chitosan nanoparticles were prepared via ionic gelation with tripolyphosphate, and they were loaded with VM. The focus of this paper was to propose the Spanish broom as alternative to cotton in wound healing bandages. However, the paper also highlights the ability of the Spanish broom to retain VM-loaded chitosan NPs and to achieve suitable VM release [46].

VM encapsulation in poly(lactic-co-glycolic acid) and polylactic acid pH-sensitive polymers were experimented on in order to overcome VM drawbacks, such as the strong pH-dependent charge, tendency to aggregate, low bioavailability, and poor cellular uptake, and to deliver VM specifically at a slightly acidic pH corresponding to infection sites. The NPs were prepared using a simple and reproducible method, establishing strong electrostatic interactions between VM and the (co)polymers’ end groups with VM payloads up to 25 wt%. The drug-loading mechanism was investigated using solid-state nuclear magnetic resonance spectroscopy. The NPs remained stable during storage and did not release the incorporated drug at a neutral pH, whereas slight acidification of the medium triggered the rapid release of VM. These compartmentalized NPs have potential applications for controlled VM release at infection sites with a local acidic pH [61].

As far as VM release from polymer NPs, it strongly depends on the type of polymer, its charge, and interaction with VM, which has a strong pH-sensitive dependent charge. While the Higuchi model with Fickian diffusion is represented, also the non-Fickian release of VM from trimethylchitosan NPs was demonstrated by Xu and colleagues [62].

Exploiting differences at the infectious site is becoming more attractive for the development of a new smart therapy that requires the drug to remain inactive in the physiological environment and exert its effect triggered by cues at the disease site. For this aim, a smart delivery system for on-demand antibiotic release, by exploiting the bacterial microenvironment at the infection site, was studied [63].

A vancomycin-encapsulated pH-responsive solid lipid nanoparticle (SLN) system was fabricated using acid-cleavable lipid SA-3 M (abbreviated as VM-FB_SA-3M_SLNs). The nanoparticle showed long-term stability up to 3 months under neutral pH. Under the acidic conditions of bacterial infection, SA-3 M was degraded to release the vancomycin cargo. A further in vivo study using a mouse wound infection model demonstrated the enhanced antimicrobial activity and reduced inflammatory responses of wound sites treated with vancomycin-loaded SLN [64].

Mohapatra and colleagues [65] developed a magnetic-stimulus-responsive vancomycin drug delivery system based on chitosan microbeads embedded with magnetic nanoparticles. In this study, they demonstrated that this DDS has the potential to burst-release higher amounts of drugs on multiple instances of the magnetic stimulus, several hours or days (16 days) apart as needed, and thus might enable us to maintain or control drug concentrations in the targeted infectious location.

Mas, N.; and co-workers [66] developed mesoporous silica nanoparticles (MSNs) able to release the vancomycin in the presence of bacteria. MSNs were functionalized with N-[(3-trimethoxysilyl)propyl] ethylendiamine triacetic acid trisodium salt (TMS-EDTA) and capped with the cationic polymer ε-pLys via electrostatic interactions with the negatively charged NPs surface. The presence of bacteria (*E. coli*, *Salmonella typhi*, and *Erwinia carotovora*) triggers pore uncapping, due to the adhesion of the ε-pLys gatekeeper with the negatively charged bacterial wall, which allows for the release of the entrapped cargo.

### 5.2. Inorganic Nanoparticles

Vancomycin has been conjugated with inorganic nanoparticles, and various papers demonstrate enhanced antibacterial activity for VM-immobilized nanoparticles, by lowering the MIC with respect to free VM, for all tested bacteria. For example, VM-conjugated gold nanoparticles outperformed VM alone by 64-fold [67]. Rashid and colleagues conjugated VM with oxide magnetite (Fe_3_O_4_) nanoparticles through a ligand exchange technique that employs the catechol group of Dopamine to anchor DOPA to iron oxide nanoparticles. The surface of the resulting Fe_3_O_4_/DOPA nanoparticles contains amino (–NH_2_) groups that are conjugated with VM via a coupling reaction between the –NH_2_ group of dopamine and the –COOH group of vancomycin [68]

Hagbani and colleagues [55], aimed to improve the VM antibacterial potential through gold nano-formulations. They employed a simple one-pot approach to synthesize VM-loaded gold nanoparticles (V-GNPs), utilizing vancomycin’s reducing abilities to create V-GNPs from gold ions. UV-visible spectroscopy confirmed the synthesis of V-GNPs, revealing a surface plasmon resonance peak at 524 nm. Transmission electron microscopy revealed a nanoparticle size of around 24 nm, whereas dynamic light scattering estimated a hydrodynamic diameter of 77 nm. Zeta-potential measurements were used to study the stability of V-GNPs, which revealed a zeta potential of −18 mV. The study found that VM-functionalized gold nanoparticles may be a feasible nano-platform for fighting bacterial resistance.

Sharma, D. and Chaudhary, A. [69] aimed to develop an efficient antibacterial agent through the simple, robust, and eco-friendly one-pot synthesis of GS-conjugated gold nanoparticles (G-GNPs), which served as both a reducing and stabilizing agent. The resulting nanoparticles were characterized, revealing a spherical form with a hydrodynamic diameter of about 15 nm and high stability. G-GNPs effectively inhibited the growth of Gram-positive and Gram-negative bacteria, including *E. coli* DH5α, ATCC 25922, and *S. aureus* MTCC 31601. Interestingly, the G-GNPs showed remarkable efficacy against GS-resistant *Escherichia fergusonii* ATCC 354691. The study found that the produced G-GNPs, which displayed little cytotoxicity toward the mouse myoblast C2C12 cell line, have tremendous potential against Gram-positive, Gram-negative, and drug-resistant bacteria.

Bhattacharya and Neogi (2017) reported the development of a novel antibiotic agent by coating iron oxide nanoparticles with GS. These nanoparticles were obtained via a co-precipitation approach, and their surfaces were functionalized with GS [30]. The average particle size was found to be approximately 14 nm for unmodified nanoparticles and 10 nm for modified nanoparticles. The drug-release profile of the coated NPs showed a quick burst effect followed by gradual sustained release. In vitro tests against various Gram-positive and Gram-negative bacterial strains revealed that the drug–NP combination had significant antibacterial activity. The minimum inhibitory concentration (MIC) data indicated that small amounts, such as 0.2 mg/mL of drug-capped particles, may cause around 98% bacterial death. The new aspect of the work is the drug capping of the nanoparticles, which preserves both iron oxide superparamagnetic and medical properties. This formulation has been shown to be extremely blood compatible [30].

### 5.3. Liposomes

Liposomes are microscopic, spherical vesicles made up of phospholipids, the fundamental building blocks of cell membranes. This specific characteristic makes them particularly suitable for drug delivery due to several advantages, such as biocompatibility, versatility in encapsulating both lipophilic and hydrophilic compounds, potential for target delivery, and controlled release of the encapsulated drugs.

A study by Mugabe, Azghani, and Omri found that liposome-mediated GS administration is efficient against resistant strains of *P. aeruginosa* obtained from cystic fibrosis patients. The encapsulation efficiency of all liposomal preparations ranged from 4% to 5.18% of the initial drug concentration in solution. When liposomes in different compositions (1,2-dimyristoyl-sn-glycero-3-phosphocholine, 1,2-dipalmitoyl-sn-glycero-3-phosphocholine and 1,2-distearoyl-sn-glycero-3-phosphocholine + cholesterol in 2:1) were incubated in normal human pooled plasma or PBS at 4 °C or 37 °C for 48 h, 60–70% of the encapsulated GS (from 4 to 5.18% encapsulation efficiency) was retained [70].

Atashbeyk, D.G. and colleagues [71] carried out a study on the antibacterial activity of oleic acid and GS against methicillin-resistant *S. aureus* (MRSA). The researchers discovered that the combination of GS and oleic acid had synergistic effects against MRSA. When GS was combined into liposomal forms, its minimum inhibitory concentration (MIC) values were reduced 15-fold, whereas gentamicin and oleic acid reduced the MIC values 27-fold. The liposomal combination inhibited and killed bacteria more effectively than VM (commonly used to treat MRSA), making it the most efficient compound in the time–kill testing. The liposomal formulations can improve antibacterial action, lower the effective concentration required, and cause rapid bacterial inhibition.

Nicolosi, D. and colleagues [72] carried a study on the antibacterial properties of VM encapsulated in fusogenic liposomes, commonly known as SUVETs. The method of preparation includes encapsulating VM in these liposomes. Fusogenic liposomes have a positive charge, which improves Gram-negative bacterial targeting and allows for close membrane interactions via charge attraction. This encapsulation procedure improved vancomycin’s capacity to enter Gram-negative bacteria, which was previously ineffective due to its inability to pass the bacterial cell membrane. As a result, VM’s antibacterial activity was increased by including Gram-negative bacteria. This novel strategy may lead to more effective therapies for infections caused by Gram-negative bacteria that were ineffective previously.

Abrishami, M. and colleagues [73] carried out a study to assess the in vivo efficacy of a liposomal formulation of VM against methicillin-resistant *S. aureus* (MRSA) in rabbits. The rabbits received a liquid culture medium containing MRSA, and after 48 h, the eyes were treated with a nano-liposomal formulation and free VM. The rabbits were euthanized at predetermined intervals of 12, 24, 48, 96, and 144 h following injection. The antibacterial activity for different VM formulations was assessed using the time-killing method. The liposomal VM had a zeta potential of 29.7 mV, mean liposome size of 381.93 ± 30.13 nm, and 47% encapsulation efficiency. The results of time-killing studies indicated that the liposomal formula was more effective than VM in a free form. Thus, it was concluded that the nanoliposomal formulation is a significant antibacterial agent to combat infectious endophthalmitis.

Vancomycin-loaded PEGylated liposomes (PEG-VM-lipos) were effective in reducing vancomycin-induced kidney damage. The study performed by Joshi and colleagues tested, at first, PEG-VM-lipo in vitro cytotoxicity on kidney cells with PEG-VM-lipo and discovered that it was less toxic than conventional VM. Secondly, male adult rats were given either PEG-VANCO-lipo or VM HCl. Plasma VM concentrations and KIM-1, an injury biomarker in urine, were compared. On day three, the PEG-Vanco-lipo group had less VM in their urine and kidneys, as well as KIM-1, than the VM group. On the first and third days, the VM group had significantly lower plasma VM concentrations than the PEG-VM-lipo group [74].

Strategies to improve VM loading into liposomes have been used. In example Sybil Obuobi and colleagues designed and developed a nanostructured hybrid system wherein nucleic acid nanogels are caged within a liposomal vesicle for VM intracellular delivery. The authors exploited the different charges of DNA nanogels and VM to improve VM loading into liposomes made from pure soy phosphatidylcholine. The binding affinity between the DNA nanostructures and VM significantly increased the antibiotic loading and resulted in a relatively slower release profile than the liposomal or nanogel formulations alone. DNA nanogels encapsulated in liposomal vesicles were proposed as a universal loading platform for the intracellular delivery of antibiotics [75].

### 5.4. Dendrimers

Among the promising nanocarriers, dendrimers have emerged as a class of versatile and tunable materials with unique properties that make them well-suited for delivering GS and VM [76]. Dendrimers are well-defined, highly branched, and monodisperse synthetic macromolecules with a core, branching units, and peripheral functional groups [77]. A recent study was conducted by Sheykhloo, H. and colleagues [78] to develop GS-conjugated poly(amidoamine) (PAMAM) dendrimers to improve the therapeutic efficacy of GS against *P. aeruginosa*. Gentamicin-presenting dendrimers were created by utilizing MAL-PEG3400-NHS as a redox-sensitive linker to attach GS to the surface of G4 PAMAM dendrimers. Gentamicin linked to the surface of PAMAM dendrimers exhibited three times the antibacterial activity of non-conjugated GS. The PAMAM-GS dendrimers were found to be at least 13 times more effective against biofilms than GS under normal conditions.

Chosy, M.B. and colleagues [79] carried out a study to expand the antibiotic potential of VM. The authors were inspired by previous studies on cell-penetrating guanidinium-rich transporters and introduced VM conjugates that effectively eradicate Gram-positive biofilm bacteria, persistent cells, and VM-resistant enterococci. They also reported, for the first time, VM conjugates with dendrimer-displayed guanidinium groups exhibiting superior efficacy and breadth. These conjugates, V-r8 and V-R, demonstrated the best activity as single broad-spectrum compounds effective against ESKAPE pathogens. The study introduces a new class of broad-spectrum VM derivatives and outlines a general strategy to enhance or expand antibiotic performance through combined mode-of-action and function-oriented design studies.

### 5.5. Micelle-Based Drug Delivery

Micelles are self-assembled, amphiphilic colloidal aggregates that encapsulate hydrophobic drugs, protecting them from degradation and aiding their transport in the body’s aqueous environment. Their properties, such as size, biocompatibility, and controlled release, make them significantly attractive for drug delivery. They can target specific tissues, decrease potential drug toxicity, and allow for sustained and targeted drug delivery. Several studies have shown the potential of micelle-based delivery in overcoming challenges associated with conventional antibiotic administration and specifically with GS and VM.

Xia, W. and colleagues [80] carried out a study intended to create a novel dual-drug delivery system using mesoporous bioactive glass/polypeptide graft copolymer nanomicelle composites. The researchers utilized water-soluble GS and fat-soluble naproxen as models. Each of these drugs was contained within mesoporous bioactive glass and polypeptide nano-micelles, respectively. The release of these drugs was subsequently investigated at various pH levels. The study showed the pH-controlled release of individual drugs. Gentamicin was predominantly released from the mesoporous bioactive glass in an acidic environment, whereas naproxen was rapidly released from the polypeptide nano-micelles in an alkaline environment. This pH-controlled release implies that individual drug release can be influenced by the environmental pH. This case study sheds light on the development of dual-drug delivery systems, showing the potential of mesoporous bioactive glass/polypeptide graft copolymer nano-micelle composites in improving therapeutic efficacy while reducing adverse effects. The pH-controlled release mechanism is a potential technique to target and sustain medication delivery.

Vancomycin-loaded micelles were discovered to be more efficient than free vancomycin in treating MRSA infections in mice. It has been reported that micelle-encapsulated vancomycin had better targeting and penetration into infected tissues, resulting in higher bacterial death and a lower bacterial load compared to the free drug. Furthermore, the micelle-based formulation showed lower systemic exposure, potentially reducing the likelihood of adverse effects.

Chen, M. and colleagues [81] used, as a base material for micelle carrier preparation, amphiphilic poly(ethylene glycol)-poly(ε-caprolactone) (PECL) copolymers conjugated with VM as targeting ligands via pH-cleavable hydrazone bonds (VM-hyd-PECL). Then, ciprofloxacin (CIP) was encapsulated to produce VM-hyd-PECL/Cip micelles showing an average size of 77 nm and CIP loading of 4.5%. The poly(ethylene glycol) shells and the expansion of VM moieties on the micelle surface were intended to improve blood circulation and bacterial recognition. The study found that deshielding VM shells in an acidic environment disturbs the hydrophobic/hydrophilic equilibrium, resulting in increased micelle sizes. This allows lipase to degrade poly(ε-caprolactone) near the infection site, releasing encapsulated CIP for bacterial destruction. Micelle treatment increased the survival rate of Pseudomonas aeruginosa-infected mice while decreasing bacterial burdens and alveolar damage in the lungs when compared to free drugs and micelles without VM moiety inoculation. Three doses of VM-hyd-PECL/Cip micelles improved animal survival, reduced bacterial colonization in the lungs, and nearly restored the normal alveolar microstructure. This case study illustrates an approach for improving bacterial targeting of micelles using an antibiotic (VM) and sequentially triggering the release of antibiotics (VM and CIP) at the infection site. These case studies highlight the potential of micelle-based delivery systems to overcome limitations associated with conventional antibiotic administration. By improving the solubility, bioavailability, and targeting, micelles offer a promising system for enhancing the efficacy and safety of antibiotics in the fight against bacterial infections, including those caused by MDR bacteria.

However, further research is required to explore the long-term safety and efficacy of micelle-based drug delivery systems and translate these promising preclinical findings into clinical applications. Future investigations should focus on optimizing the micelle design, conducting large-scale clinical trials, and addressing regulatory considerations to ensure the safe and effective translation of this technology into clinical practice.

### 5.6. Carbon-Nanotube (CNT)-Based Drug Delivery

This section addresses the suitability of CNTs for delivering antibiotics and their potential benefits in overcoming challenges associated with conventional antibiotic administration. Carbon nanotubes are cylindrical nanostructures formed by rolling a single sheet of graphene into a seamless tube. They possess a high aspect ratio, exceptional strength, stability, tailorable functionality, and unique electrical properties. These attributes make them suitable for various applications, including drug loading, targeted drug delivery, and triggered drug release [82,83,84].

Liu, C. and colleagues [85] aimed to overcome the limitations of multi-walled carbon nanotubes (MWCNTs) in infection resistance due to aggregation into the polymer matrix and weak bactericidal properties. The researchers created VM-hydrochloride-modified MWCNT (VM-MWCNT) by reacting the carboxyl group of MWCNT with VM’s amide group. The Van-MWCNT was absorbed onto TPU electrospun nanofibers (TPU/Van-MWCNT) via ultrasonication. The preparation technique was non-toxic and utilized water as a green solvent. The approach successfully minimized the aggregation of VM-MWCNT into electrospun nanofibers. The study found that VM-MWCNT has a lower minimum inhibitory concentration (MIC) against *S. aureus* compared to MWCNT. The TPU/Van-MWCNT showed remarkable antibacterial characteristics, indicating possible uses in wound dressings.

Al Thaher and colleagues [86] studied a novel PMMA bone cement to prevent prosthetic joint infections (PJIs). The goal was to investigate GS release from carbon nanotubes (CNTs) embedded in polymethyl methacrylate (PMMA) used as bone cement over several weeks to provide post-surgery prophylaxis against PJIs. Gentamicin was examined at various CNT concentrations, either as a powder or preloaded on carboxyl functionalized CNTs. The findings revealed that CNT-loaded bone cements released gentamicin for several weeks longer than GS-containing bone cement. Furthermore, the addition of CNT increased the amount of GS released without impairing the nanocomposite’s mechanical and antibacterial properties. The bone cement implemented with CNT performed similarly to the corresponding powder-containing cement in terms of cytotoxicity. This novel technique could be effective in preventing PJIs in orthopedic procedures.

## 6. Nanosized DDS Interactions with Bacterial Membranes

As reported in the examples above, nanosized DDSs have been an area of significant research interest due to their potential to improve the efficacy and specificity of antimicrobial treatments. However, the mechanism of interaction of these nanosized DDSs with bacterial membranes still remains a critical aspect to explain their function. The main mechanisms involved the interaction between DDS and bacterial membranes are electrostatic interactions, hydrophobic interactions, and ligand–receptor binding [87,88].

Gram-negative bacterial membranes, due to the presence of phospholipids and lipopolysaccharides (LPS), express a negative charge; exploiting electrostatic interaction mechanisms can be used to design DDSs with positive charges to enhance electrostatic attraction, improving adhesion and interactions with bacterial membranes [89].

Bacterial membranes are composed of a lipid bilayer that has hydrophobic regions. DDSs with hydrophobic surfaces or hydrophobic drug molecules can integrate more effectively into these regions, facilitating drug delivery. Moreover, is important to also consider the differences in compositions and structures of Gram-positive and Gram-negative membranes. The wall of Gram-positive bacteria consists of a thick peptidoglycan layer (20–80 nm), and unlike Gram-negative bacteria, Gram-positive bacteria do not have an outer membrane. Gram-negative bacteria have an outer membrane composed of lipopolysaccharide (LPS), phospholipids, and proteins, with a thinner peptidoglycan layer (2–7 nm) located in the periplasmic space between the inner and outer membranes [90].

Finally, for a more specific and selective interaction, DDSs can be functionalized with ligands that can bind receptors on bacterial surfaces (e.g., antibodies, peptides, sugars) enhancing targeting and uptake. In the case of Gram-positive targeting, functionalizing DDSs with molecules that can interact with teichoic and lipoteichoic acids can enhance adherence and uptake. For Gram-negative bacteria, a strategy to overcome the limiting step of a thicker membrane could be design-engineered DDSs to utilize porins or interact with LPS for entry or to disrupt the outer membrane to gain access to the periplasmic space [91].

**Figure 6:**
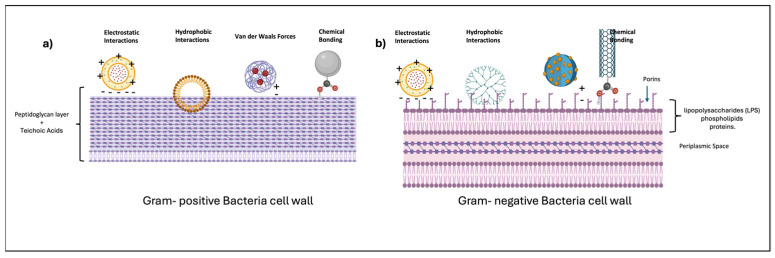
Nanosized DDS interactions with bacterial membranes: (**a**) Gram-positive and (**b**) Gram-negative.

Figure 6 summarizes nanosized DDS interactions with bacterial membranes (Gram-positive and Gram-negative).

## 7. Discussion

Nanoparticle-based drug delivery systems provide attractive alternatives for improving treatment efficacy, overcoming resistance, and limiting the unwanted effects of antibiotics. In this comprehensive review, we explored various nanosystems for GS and VM delivery. Polymeric NPs are extensively studied because of their biocompatibility, configurable characteristics, and sustained release profiles. For GS and VM the most studied polymer NPs are poly(lactic-co-glycolic acid) (PLGA). It provides the advantages of high drug-loading capacity, controlled release, and enhanced antibacterial activity [12]. In the case of inorganic nanoparticles, silver nanoparticles (AgNPs) and gold nanoparticles (AuNPs) are extensively studied for GS and VM. They offer the advantage of greater antibacterial activity suitable drug loading and stability [9]. Liposomes, lipid-based vesicles, are versatile drug carriers providing the advantages of the improved bioavailability, controlled release, and high drug-loading capacity that has been widely exploited for GS and VM. There exists a variation in drug-encapsulating efficiency for both antibiotics depending the type of liposomes and the experimental parameters. For instance, the encapsulation efficiency for gentamicin was reported to be 25.7 ± 1.0% for DPPC/chol vesicles [92], 2.9% for neutral, DPPC-Chol (55:45), 9.3% for anionic DOPE–N-succinyl-DOPE–PEG (69:30:1) [93], 1.8% to 43.6% (1.8% ± 0.15% for neutral liposomes, 37.2% ± 0.46% and 43.6% ± 0.65% for negatively charged liposomes) [31], and 4.51 ± 0.54% for neutral liposomes (DMPC-Chol 2:1) [94]. For VM, the encapsulation efficiency also varies as it was 8.84 ± 2.1% for neutral liposomes [95], 9 ± 2% (neutral) and 12 ± 3% (PEGylated) [96], 78.66% (containing propylene glycol as a permeation enhancer) [97], and 9% (for DCP) and 20% (for DMPG) [98]. Dendrimers also offer advantages of tailored surface properties, but their toxicity needs to be studied well. Among them, PMAM dendrimers are most commonly used. Micelles also improve drug solubility and stability with controlled release. Carbon nanotubes offers unique physiochemical properties, but their toxicity needs to be considered as well.

Although, PLGA NPs, AgNPs, liposomes, PAMAM dendrimers, polymeric micelles, and functionalized CNTs are potential nanosystems for GS and VM administration, liposomes stand out for their high drug loading, controlled release, and increased bioavailability, also due to their ability to cross bacterial membranes via fusion with them. Liposomes show strong efficacy against bacterial biofilms (due to their structure, they reach deeper biofilm layers), and also the concern of toxicity is very less for liposomes. To the best of our knowledge, there are no drug products on the market made from liposomal GS or VM, while there are, in the market, drug products made from liposomes loaded with amikacin and tobramycin. These findings mean that liposomal formulations loaded with GS and VM still need to be optimized.

From an industrial standpoint, liposomes are one of the most commonly employed nanodrug delivery technologies. They have been widely researched and are currently employed for various drugs that are approved by the FDA. This is because they are biocompatible, can encapsulate both hydrophilic and hydrophobic medicines, and are easy to produce on a large scale. Liposomes can be made utilizing a variety of processes, including thin-film hydration, reverse-phase evaporation, and detergent removal, which are easily scaled up for industrial manufacturing. However, it is vital to note that the choice of nanodrug delivery system can rely on several aspects, including the nature of the drug, the targeted delivery site, and the specific patient need [99,100,101,102].

Challenges and future directions in nanoparticle-based drug delivery systems concern the development of cost-effective and scalable manufacturing processes for antibiotic-loaded nanoparticles. With a view to clinical application, ensuring the stability of nanoparticles during storage and transportation is critical. Eventually, from a regulatory point of view, comprehensive studies on the long-term safety and potential immunogenicity of nanoparticle systems are required. When deciding on the best solution, researchers should examine both safety profiles and particular application requirements. Future advances in nanoparticle–drug conjugates provide considerable promise for addressing antibiotic resistance.

## 8. Conclusions

The growing burden of infectious diseases, along with increasing pathogen resistance to current therapeutic therapies, necessitates novel drug delivery techniques. Nanoparticles’ unique physicochemical features provide a viable solution with respect to standard drug formulation limits, by increasing bioavailability, facilitating drug-controlled release, and permitting targeted administration. This not only maximizes therapeutic efficacy but also reduces the likelihood of resistance development.

However, the journey to successful nanodrug delivery systems is filled with obstacles, ranging from the intricacies of microbial resistance to the difficulties posed by biofilm-associated and intracellular infections. Nevertheless, the nanosized systems still need optimization as far as their manufacturing technique and drug loading is concerned. Some measures that can complement the use of nanosized drug delivery devices include enhanced diagnostics, judicious antibiotic use, novel antibiotics discovery, and combination therapy.

In conclusion, while the road ahead is challenging, the potential of nanosized drug delivery devices to improve the efficacy of antibiotics, such as gentamicin and vancomycin, is promising. Continued research and development in this subject are critical to realize its promise and reverse the tide in the ever-changing war against infectious diseases. The goal is to create a synergistic platform that targets a wide spectrum of infectious risks, ultimately improving patient outcomes and changing the future of healthcare.

## Figures and Tables

**Table 1:** Examples of publications on gentamicin-loaded polymer and inorganic nanoparticles.

Nanosized Delivery System	NP Preparation Method	Target Bacterial Strain	Reference
Gentamicin-loaded CaCO_3_ nanoparticles	Microemulsion	*Staphylococcus aureus*	[28]
Silica–gentamicin nanohybrids	Base-catalyzed precipitation	*Bacillus subtilis*, *Pseudomonas fluorescens*, *E. coli*	[29]
Gentamicin coated iron oxide nanoparticles	Co-precipitation	*S. aureus*, *E. coli*, *P. aeruginosa*, *Bacillus subtilis*	[30]
Gentamicin-loaded liposomes	Dehydration–rehydration	*P. aeruginosa*, *K. oxytoca*	[31]
Gentamicin nanoparticles	water-in-oil-in-water	*K. pneumoniae*	[32]
Gentamicin-loaded CaCO_3_ nanoparticles	Carbonization	*B. subtillis*	[33]
Gentamicin-loaded chitosan nanoparticles	Ionic gelation	*Brucella melitensis*	[34]
Gentamicin-loaded silk/nanosilver composite	Chemical synthesis	Methicillin-resistant *S. aureus* (MRSA)	[35]
Gentamicin sulfate-loaded PLGA nanoparticle	Double emulsion solvent removal	*P. aeruginosa*, *S. aureus*	[36]
Gentamicin-loaded proanthocyanidin–chitosan composite nanoparticles	Ionic gelation	*E.coli*, *S. aureus*, *P.aeruginosa*	[37]
Gentamicin-loaded PLGA nanoparticles	Double emulsion evaporation	*E. coli*	[38]
Gentamicin nano gel	Sol-gel application	*E.coli*, *St. epidermidis*	[39]
Gentamicin-loaded chitosan/folic acid-based carbon quantum dots	Hydrothermal technique	*E. faecalis*, *P. aeruginosa*, *S. mutans*, *S. aureus*, *K. pneumoniae*, *E. coli*	[40]
Gentamicin–ascorbic acid-loaded chitosan nanoparticles	Ionotropic gelation	*S. aureus*, *P. aeruginosa*	[41]
Gentamicin-loaded PLGA/polyurethane/poly(ethylene oxide) nanoparticles	Double emulsion solvent evaporation	*E. coli*	[42]
Gentamicin-coupled gold nanoparticles (G-GNPs)	Sol-gel method	*E. fergusonii*	[12]
Gentamicin-loaded PEG-PLGA/PLGAH nanoparticles	Solvent precipitation	*P.aeruginosa*, *S. aureus clinical strains*	[43]

**Table 2:** Examples of publications on vancomycin-loaded polymer and inorganic nanoparticles.

Nanosized Delivery System	NP Preparation Method	Target Bacterial Strain	Reference
Functionalized nanoparticles of α-norbornenyl-ωvancomycin poly(ethylene oxide) macromonomers	Ring-opening metathesis polymerization	Methicillin resistant *S. aureus*	[44]
Vancomycin-functionalized gold and silver nanoparticles	Chemical synthesis	Methicillin-resistant *S. aureus* (MRSA)	[45]
Vancomycin-loaded chitosan nanoparticles	Ionic gelation	*S. aureus*	[46]
Vancomycin-loaded N-trimethyl chitosan nanoparticles	Chemical synthesis	*S. aureus*	[47]
Vancomycin and Cefazolin-loaded lipid nanoparticles	Reverse phase evaporation	Methicillin-resistant *S. aureus* (MRSA)	[48]
Vancomycin–PLGA-conjugated nanoparticles	Double emulsification-solvent evaporation	*S. aureus*, *P. aeruginosa*	[49]
Vancomycin-loaded silver nanoparticles	Chemical synthesis (reduction)	*S. aureus*, *E. coli*	[50]
Vancomycin-loaded iron oxide nanoparticles	Thermal decomposition	*Clostridium difficile*	[51]
Vancomycin-loaded PLGA nanoparticles	Double emulsion solvent evaporation	*S. aureus*	[52]
Vancomycin-conjugated gold nanoparticles	Chemical synthesis	*S. aureus*, *E. coli*	[53]
Vancomycin-loaded PLGA nanoparticle	Water-in-oil double emulsion	*S. aureus*	[54]
Vancomycin-functionalized gold nanoparticles (V-GNPs)	One pot synthesis	*E.coli*, *Klebsiella oxytoca*, *S. aureus*, *P. aeruginosa*	[55]
Vancomycin-loaded PLGA nanoparticles	Emulsification-solvent evaporation	*Enterococcus faecalis*	[22]
Vancomycin-loaded chitosan nanoparticles	Ionotropic gelation	*S. aureus*	[56]
PLGA nanoparticles loaded with vancomycin and conjugated with lysostaphin (PLGA-VAN-LYS)	Double emulsion evaporation	*S. aureus*	[57]
